# Is overnight fresh juice drinkable? The shelf life prediction of non-industrial fresh watermelon juice based on the nutritional quality, microbial safety quality, and sensory quality

**DOI:** 10.29219/fnr.v64.4327

**Published:** 2020-06-09

**Authors:** Tingting Ma, Jiaqi Wang, Haoli Wang, Tian Lan, Ruihao Liu, Tian Gao, Wanyi Yang, Yuan Zhou, Qian Ge, Yulin Fang, Xiangyu Sun

**Affiliations:** 1 College of Food Science and Engineering, College of Enology, Viti-viniculture Engineering Technology Center of State Forestry and Grassland Administration, Shaanxi Engineering Research Center for Viti-Viniculture, Northwest A&F University, Yangling, China; 2 China Wine Industry Technology Institute, Yinchuan, China

**Keywords:** quality assurance, non-industrial fresh watermelon juice, microbial safety quality, nutritional quality, sensory quality, shelf life

## Abstract

**Objective:**

The aim of this study was to obtain the time range of non-industrial fresh watermelon juice (FWJ), which is widely used in the catering industry under different storage conditions, with safe-drinkable quality, and the drinking time range of fresh juice with good nutritional quality and sensory quality.

**Method:**

The quality of non-industrial FWJ was audited by assessing the shelf life of non-industrial FWJ through microbial safety, nutritional, and sensory quality investigating during 24 h of storage at 4, 25, and 37°C.

**Results:**

According to the microbial safety quality, the safe drinking time of FWJ was within 12, 4, and 4 h when stored at 4, 25, and 37°C, respectively. Based on the nutritional and sensory quality, FWJ was drinking with good quality within 2 h, and with just acceptable quality for no more than 4 h when stored at 4 or 25°C. Electronic nose and gas chromatography–mass spectrometry (GC–MS) could effectively distinguish and identify the changes in volatile components in FWJ under different storage conditions.

**Conclusion:**

It is a feasible method to predict the shelf life of non-industrial FWJ by this method, and hence to guarantee non-industrial FWJ being drinking with safety and health, and it might be used in many other fresh juice shelf life predictions.

**Figure:**
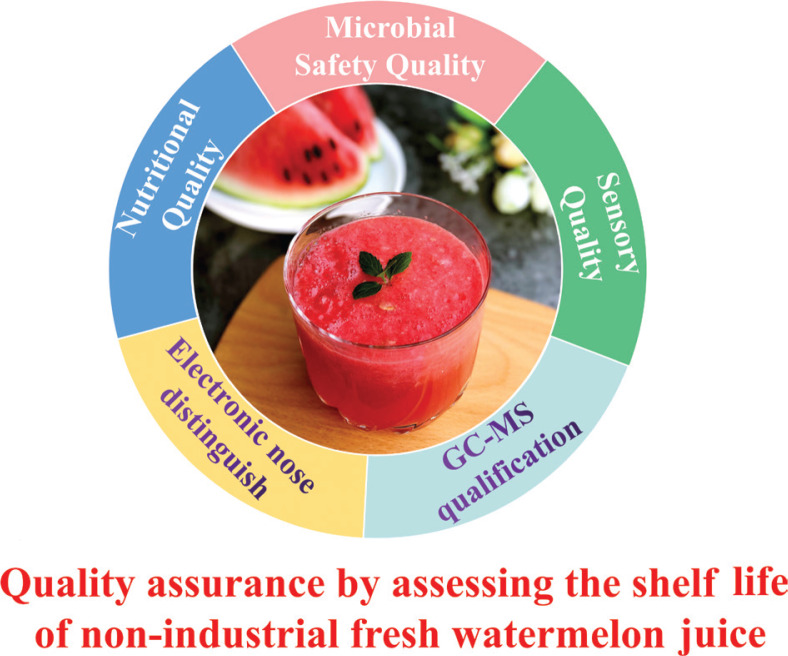

## Popular scientific summary

- The shelf life of fresh watermelon juice were predicted.
- The safe drinking time of FWJ was within 12, 4, and 4 h when stored at 4, 25 and 37°C.
- The drinking time of FWJ with good quality was within 2 h when stored at 4°C or 25°C.
- The FWJ still had acceptable quality when stored at 4°C or 25°C for no more than 4 h.
- E-nose and GC-MS could distinguish and identify the changes in volatile in FWJ.

With the improvement in the food consumption level and dietary habits, people are increasingly pursuing natural, nutritious, and healthy food (1). Fresh juice not only provides rich nutrients, a pleasurable taste, and an attractive color, but it also has no additives and is pure, natural, and minimally processed. Therefore, in recent years, the consumption of fresh juice has significantly trended upward, and fresh juice is gaining much popularity among customers throughout the world; it has gradually become the new favorite of the beverage industry (2, 3). Non-industrial fresh watermelon juice (FWJ) is one of the most common fresh juices in the market; it is an excellent product to quench thirst and relieve the heat in summer and the tropics. Non-industrial FWJ, which contains several minerals (Mg, K, Ca, and Fe), vitamins (A, B, C, and E), specific amino acids (citrulline and arginine), and various antioxidants (polyphenol and lycopene), is highly nutritious (4, 5). Moreover, due to its pleasant taste and attractive color, FWJ has become a popular health functional beverage.

China’s Food and Drug Administration recommends that non-industrial fresh juice should be consumed as soon as possible. However, for both homemade fresh juice and catering services non-industrial fresh juice, timely drinking cannot be guaranteed due to many reasons. For example, a juice shop prepares fresh juice in advance and waits for customers to buy it within a period of time; a housewife prepares too much juice to drink at a single time and drinks the remainder the next day; and a juice shop prepares too much juice to sell in 1 day and then stores the remainder to be sold the next day (6). In addition, compared with industrialized juice, non-industrial fresh juice, including non-industrial FWJ, is usually drank directly after juicing without any sterilization treatment, which results in the problem that bacteria are easily bred and cause microbial contamination during storage and retail (2, 7–9). In addition, as non-industrial FWJ does not contain any food additives, its sensory index and nutritional and functional indicators will change significantly with the extension of storage time; it is easy to cause sensory deterioration and nutritional loss if stored improperly or for a longer storage time (2, 10). At present, many countries have not yet developed the relevant hygiene standards and quality standards for non-industrial fresh juice, which brings great inconvenience to the management of the freshness and microbial safety of non-industrial fresh juice. Therefore, it is very important and meaningful to analyze and master the quality change rules of various non-industrial fresh juices under different storage conditions and obtain the shelf life of non-industrial fresh juice with safe-drinkable quality and good nutritional quality and sensory quality. Only in this way, the production, management, and consumption of non-industrial fresh juice can be scientifically guided, and a good and safety quality for consumers can be assured.

Based on the aforementioned information, in this study, the dynamic changes in microbial indexes of non-industrial FWJ, which are widely used in the catering industry under different storage conditions, were measured and analyzed, aiming to obtain the time range of non-industrial FWJ with safe-drinkable quality. In addition, the dynamic changes in sensory, nutritional, and functional indicators of the juice under different storage conditions were also investigated, to obtain the drinking time range of fresh juice with good nutritional quality and sensory quality. Furthermore, a new method for evaluating the shelf life and the quality of fresh juice was also established. This study can provide certain guidance for the quality assurance of sale and management in non-industrial FWJ in catering as well as provide some basic data and a theoretical reference for consumers to judge the quality of non-industrial FWJ.

## Materials and methods

### Samples and chemicals

Jingxin No. 1 fresh watermelons (fully ripened, no mildew, no pests, or diseases) were purchased from a local market (Trust-mart Chain Supermarket, Yangling, China) to produce non-industrial FWJ. The watermelon was peeled, cut into small cubes, and crushed using a juice extractor (H-AE-DNBI19, Seoul Hurom Co. Ltd., Seoul, Korea). The juice extractor, cutter, and kneading board were thoroughly sterilized by UV or heating. The juicing process was carried out in accordance with standard operating specifications.

Non-industrial FWJ was poured into pasteurized glass bottles and stored at 4, 25, and 37°C, which was tried to simulate the refrigerated storage temperature, the indoor temperature, and the summer high temperature, respectively. The changes in the microbial index and sensory, nutritional, and functional indexes were measured after 0, 2, 4, 8, 12, and 24 h, and the sensory characteristics of non-industrial FWJ under different storage conditions were measured by an electronic nose and gas chromatography–mass spectrometry (GC–MS).

All standards, including gallic acid, ascorbic acid, 1,1-diphenyl-2-picrylhydrazyl (DPPH), the Folin–Ciocalteu reagent, 6-hydroxy-2,5,7,8-tetramethylchroman-2-carboxylic acid (Trolox), and 2-octanol, were purchased from Sigma-Aldrich (St. Louis, MO, USA). Sodium acetate trihydrate, FeCl_3_·6H_2_O, 2,4,6-tripyridyl-S-triacine (TPTZ), ethanol, glacial acetic acid, HCl, Trolox, and anhydrous sodium carbonate were purchased from XinFang Chemical Reagent Co., Ltd. (Yangling, Shaanxi, China).

### Microbial analysis

Non-industrial FWJ is rich in nutrition, and it easily breeds spoilage and pathogenic microorganisms. Hence, the aerobic bacterial count (TBC) and the presence of *Escherichia coli*, molds and yeasts, were selected as microbial detection indicators in non-industrial FWJ under different storage conditions. Using the plate counting method to determine the aerobic bacterial count and the amount of molds and yeasts and using the most probable number (MPN) method for enumeration of *E. coli*, a volume of 1 mL of juice sample was mixed with 9 mL of sterile saline solution (NaCl 0.9%) and continuously diluted (10^-1^~10^-6^) for the microbial count in triplicate. Nutrient agar medium was used for detecting the aerobic bacteria, and the plates were incubated at 37°C for 48 ± 2 h. The enumeration of total molds and yeasts was carried out on Rose Bengal agar medium incubated at 25°C for 5 days. *E. coli* was were detected by lauryl sulfate peptone broth and brilliant green lactose bile broth. The plates were incubated at 36°C for 48 ± 2 h. All media were purchased from Hope Bio-Technology Co., Ltd. (Qingdao, China). The results were expressed as log [cfu/mL].

### Nutritional and functional indicators

#### Total soluble solids, pH analysis

The total soluble solids (TSS) of the juice samples were determined as °Bx using a PAL-1 digital Abbe Refractometer (ATAGO Co., Tokyo, Japan). The pH values of the juice samples were measured using a PHS-3E pH meter (Shanghai Leici Co. Ltd., China).

#### Determination of ascorbic acid

Ascorbic acid of fresh juices was measured using the 2,6-dichlorindophenol method with minor modification (11). Aliquots of 10 mL of juice samples were placed individually in 50 mL centrifuge tubes; then, 10 mL of extracting solution (metaphosphoric acid-oxalic acid solution) was added. The mixture was treated with a vortex for 3 min, kept stable for 30 min, and then centrifuged at 8,000 *g* for 8 min using a 5804R centrifuge (Eppendorf Co., Hamburg, Germany). A volume of 10 mL supernatant was then titrated with 2,6-dichlorindophenol solution until a faint pink color was observed for 15 consecutive seconds. In addition, a blank test was carried out to eliminate interference. Ascorbic acid was calculated as follows:

Ascorbic acid (μg/mL) = [(*V*–*V*_0_) ×*T* ×*A* × 1,000]/*V*_1_

where *V* is the volume of 2,6-dichlorindophenol solution consumed in the titration of juice samples; *V*_0_ is the volume of 2,6-dichlorindophenol solution consumed by the titration blank; *T* is the titration of 2,6-dichloroindophenol solution; *A* is the dilution ratio of fresh juices; and *V*_0_ is the volume of juice samples.

#### Determination of total polyphenol content

The total polyphenol content (TPC) was determined by the Folin–Ciocalteu colorimetric method (12). The results are expressed as µg gallic acid equivalents (GAE)/mL juice (µg GAE/mL juice). The measurements were performed using a UV-1800 spectrophotometer (Shimadzu, Kyoto, Japan).

#### Analysis of the antioxidant capacity

Antioxidant capacity was determined by DPPH-free radical scavenging capacity and ferric-reducing antioxidant power (FRAP) in this study. The DPPH scavenging activity and FRAP assays were based on previously described methods with slight modifications (13). The results are expressed as μM Trolox/mL juice.

### Organoleptic indicator

#### Sensory evaluation

The method of sensory evaluation was performed according to the research of Zhang et al. (14) with slight modifications. A total of 31 untrained volunteers (16 males and 15 females) from Northwest A&F University conducted the sensory evaluation. Volumes of 200 mL of fresh juices under different storage conditions were served in a randomized order in clear glass bottles and accompanied by unsalted crackers and purified water. Volunteers evaluated the appearance, color, odor, flavor, sweetness, acidity, and overall acceptability on a 9-point hedonic scale. The hedonic scale is from 1 to 9; for each attribute, 9 points represent like it very much, and 1 point represents dislike it extremely; 5 points stand for neither like nor dislike it. After the evaluation process, the mean intensity scores of all the attributes were calculated and plotted.

### Suspension stability

As the turbidity of the juice can be divided into a precipitable part and stable part retained in the original juice during centrifugation, the absorption at 660 nm after centrifugation can be used to predict its turbidity stability during storage. The higher the light absorption value, the higher the suspension stability (15). Aliquots of 10 mL of juice samples were centrifuged at 3,000 *g*/min for 15 min, and 3 mL of supernatant was collected. The suspension stability was determined by a UV-1800 spectrophotometer (Shimadzu, Kyoto, Japan) at 660 nm, and deionized water was used as the blank.

#### Color analysis

The color attributes of watermelon juices were measured at 25°C using a Ci7600 colorimeter (X-rite, Grand Rapids, Michigan, USA) in reflectance mode. Color was expressed as L*, a*, and b* values. The total color difference (∆*E*) was calculated using the following equation, where $L_{0}^{*},a_{0}^{*},\text{ and }b_{0}^{*}$ are the control values.

$$\Delta E={\left[{\left(L^{*}-L_{0}^{*}\right)}^{2}+{\left(a*-a_{0}^{*}\right)}^{2}\;+(b^{\text{*}}-b_{0}^{*}{)}^{2}\right]}^{1/2}$$

### Detection and identification of non-industrial FWJ by an electronic nose

The electronic nose analysis was conducted using a PEN 3 electronic nose (Airsense Analytics, Schwerin, Germany) containing 10 metal-oxide semiconductors. The assay procedure was according to the research of Wang et al. (16). In total, 5 mL of fresh juice samples was placed in 20 mL vials. Testing started after equilibrating at 25°C for 10 min. Each juice sample was analyzed at least 10 times. Specific parameters of electronic nose detection were as follows: the carrier gas velocity was 300 mL/min, the detection time duration was 60 s, and the cleaning time was 240 s.

### Detection and identification of the change in volatile components in non-industrial FWJ by GC–MS

The volatile components in juice samples were analyzed by headspace solid-phase microextraction (HS-SPME) combined with GC–MS based on a previous study with minor revision (16, 17). In total, 5 mL of juice samples mixed with 1.5 g NaCl was placed in 20 mL headspace vials, 80 µL of 2-octanol (60 µL/L) was added as the internal standard, and headspace vials were sealed. Samples were equilibrated at 45°C for 30 min, and the volatile compounds were extracted from the headspace to the SPME fiber (50/30 μm; DVB/CAR/PDMS, Supelco, Bellefonte, USA) for 30 min. Then, the SPME fiber was injected into the injection port of the GC–MS system and desorbed at 250°C for 2 min.

A GC–MS-QP2010 (Shimadzu, Kyoto, Japan) was used for qualitative and quantitative analysis of the volatile compounds. A fused silica capillary column (DB-17MS; 30 m×250 μm, 0.25 μm) and helium gas (1.93 mL/min) were used for detection. The initial temperature was 40°C, and this temperature was maintained for 3 min. The temperature rose to 120°C at a speed of 4°C/min, then rose at a rate of 6°C/min to 240°C, and was maintained for 9 min. Mass spectrum (MS) conditions were as follows: the electron impact ionization (EI) ion source temperature was 230°C, the ionization energy was 70 eV, and the scanning range of MS was *m*/*z* 35–500 in the full-scan mode.

Each volatile component was identified according to the NIST 14 MS database by matching degree and retention time. Selection of substances occurred with a matching degree greater than 85% as effective aroma components. All tests were conducted in triplicate, and the relative peak areas were calculated in relation to the peak area of the internal standard area. The internal standard was 2-octanol for quantitative calculation of the content of aroma substances in different juice samples.

### Statistical analysis

Regarding the electronic nose analysis, principal components analyses were conducted by using SPSS 20.0 software (18). Other experimental results are expressed as the means ± standard deviations (SDs) of three replicates for each treatment. Correlations were calculated using linear regression. Statistical analyses were performed using DPS 7.05 software (Hangzhou, China).

## Results and discussion

### Dynamic variation rules of the microbial safety quality of non-industrial FWJ under different storage conditions

**Fig. 1:**
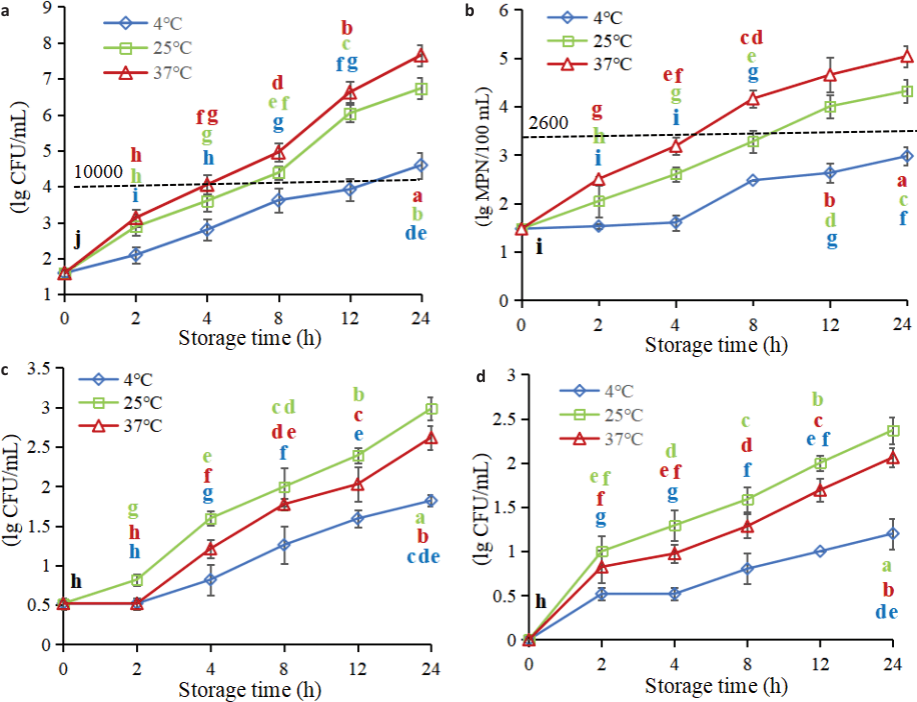
Dynamic variation rules of microbial indexes of non-industrial FWJs under different storage conditions: (a) aerobic bacterial count; (b) *Escherichia coli*; (c) mold; and (d) yeast.

Figure 1 shows dynamic variation rules of microbial indexes of FWJs under different storage conditions. The aerobic bacterial count and presence of *E. coli*, molds, and yeasts were selected as microbial detection indicators.

#### Aerobic bacterial count and amount of E. coli

The aerobic bacterial count and amount of *E. coli* in FWJ increased rapidly during the 24 h storage period (Fig. 1a, b). The two microorganism levels in juices stored at 25 and 37°C were significantly higher than those stored at 4°C (*P*<0.05). This finding indicated that storage at 4°C could significantly restrain the reproduction of the two microorganisms in the juice. The initial aerobic bacterial count and amount of *E. coli* in FWJ were 1.59 log CFU/mL and 1.48 MPN/100 mL, respectively. After 24 h of storage, the aerobic bacterial count in the juice at 4, 25, and 37°C increased to 4.59, 6.73, and 7.65 log CFU/mL, respectively, while the amount of *E. coli* increased to 2.98, 4.32, and 5.04 log MPN/100 mL, respectively. On the whole, both the aerobic bacterial count and amount of *E. coli* in the juice increased markedly with the increase in the storage temperature.

Currently, there is no widely accepted standard for the sanitary quality of fresh juice. Two local standards in China (Chinese national standard DB46/117-2008 established by Hainan province and DB33/533-2005 by Jiangsu province of China) stipulate that the aerobic bacterial count and amount of *E. coli* in fresh juice should be less than 10,000 CFU/mL (4 log units) and 2,400 MPN/100 mL (3.38 log units), respectively. Therefore, fresh juices that meet this standard can be safely consumed. Combining Fig. 1a, b, it could be concluded that the microbial safety quality of FWJ can be guaranteed for 12 h when stored at 4°C, whereas it can only be ensured for 4 h when stored at 25 or 37°C. This finding indicated that the time ranges of microbial safe drinking of the FWJ stored at 4, 25, and 37°C were 12, 4, and 4 h, respectively, and it should be emphasized that the premise of this conclusion is that the whole juicing process must be strictly in accordance with the standard operating specifications.

#### Molds and yeasts

Dynamic variation in yeasts and molds of FWJs under different storage conditions is shown in Fig. 1c, d. The results showed that the molds and yeasts in the juice exhibited a growing trend during the storage period (0–24 h). Similar to the aerobic bacterial count and amount of *E. coli*, the growth of yeast and molds was significantly inhibited when the juice was stored at 4°C. However, the difference is that the yeast and mold increased rapidly at 25°C, while the aerobic bacterial count and amount of *E. coli* grew fastest at 37°C. It is well known that the optimal growth temperature of yeast and mold is 25°C, while that of aerobic bacteria and *E. coli* is 37°C. In summary, 4°C of storage is most beneficial to ensure the microbial safety of FWJ and prolong the time range for safe drinking, and these properties could only be ensured for 12 h.

Sílvia et al. (19, 20) and Feng et al. (21) indicated that the initial aerobic bacterial count and the amount of coliform bacteria, yeasts, and molds in watermelon juice were approximately 2.5–4 log CFU/mL, which is significantly higher than what was measured in this experiment. This difference was mostly because the juice extractor, cutter, and kneading board used in this experiment were thoroughly sterilized, and the juicing process was carried out in accordance with standard operating specifications. The FWJ was poured into pasteurized glass bottles immediately after juicing for further analysis. Thus, the initial microbial loads in FWJ could be effectively controlled and reduced.

### Dynamic variation rules of nutritional and functional indicators of non-industrial FWJ under different storage conditions

#### TSS, pH analysis

**Fig. 2:**
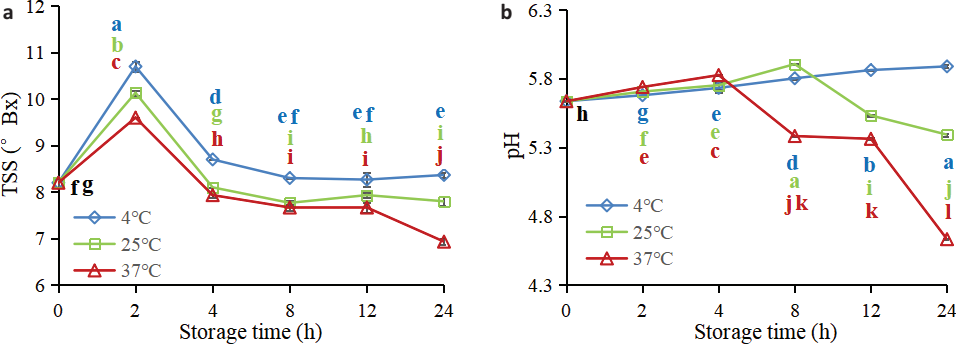
Dynamic variation rules of (a) TSS and (b) pH of non-industrial FWJ under different storage conditions.

TSS and pH are two important indicators that affect the sweetness and acidity of juice. During the whole storage period of 24 h, the TSS of FWJ stored at 4°C were significantly higher than those stored at 25 and 37°C (*P*<0.05), and the juice stored at 37°C showed the lowest TSS (Fig. 2a). Similar trends were also observed during the storage of *Arenga pinnata* juice (22) and bayberry juice (23). Thus, it is speculated that the TSS in juice negatively correlates with storage temperature. In addition, there was a noteworthy increase in TSS after 2 h of storage (*P*<0.05). Then a sharp decrease was observed during 2–24 h (*P*<0.05). This trend is mainly due to the hydrolysis of polysaccharides into monosaccharides and oligosaccharides in the early stage of storage (24). As the storage time increased, microorganisms in FWJ multiplied rapidly; TSS are used as a nutrient source for microbial fermentation, which led to a significant decrease in TSS (22, 23, 25).

As shown in Fig. 2b, the initial pH in FWJ was 5.64. The pH value of FWJ stored at 4°C was relatively stable, ranging from 5.64 to 5.89 throughout the storage period, while that of juices stored at 25 and 37°C showed a rapid downward trend after 8 and 4 h (*P*<0.05), and the pH value dropped to 5.39 and 4.63 at 24 h, respectively. Similarly, Feng et al. (21) observed an obvious decline in pH for watermelon juice during storage at 10°C. Previous studies also showed a decrease in the pH value of grape juice (25) and *A. pinnata* juice (22) with an increase in the storage period. The main reason for the decrease in pH is the increase in acidity caused by the metabolic activity of microorganisms during the storage period (25).

From Fig. 1a–d, it is clear that the microbial activity would be low at 4°C, while the microorganisms proliferate rapidly at 25 and 37°C. As microorganisms decompose carbohydrates to produce various organic acids (23), the TSS and pH of the juice stored at 4°C were relatively stable, whereas the TSS and pH of 25 and 37°C declined markedly during the later stage of storage, which was attributed to rapid proliferation of microorganisms in FWJ stored at 25 and 37°C.

#### Ascorbic acid

**Fig. 3:**
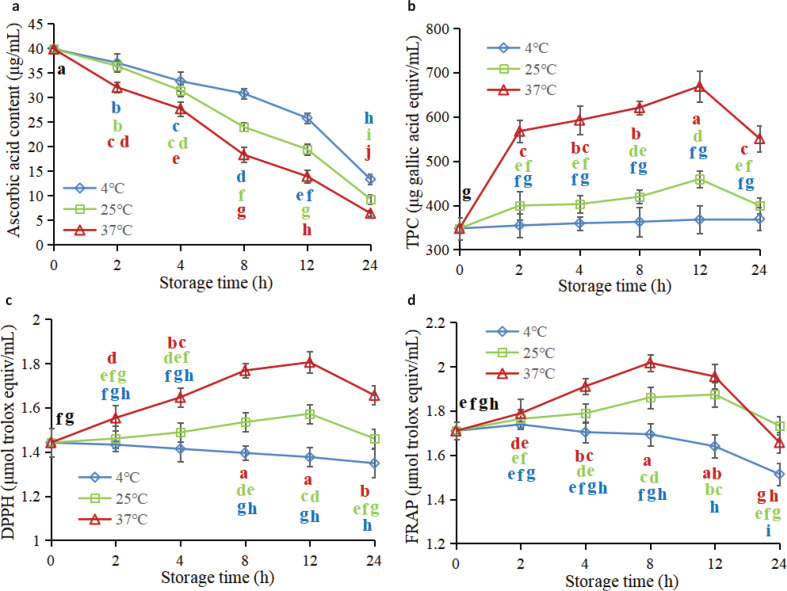
Dynamic variation rules of (a) ascorbic acid content, (b) TPC, (c) DPPH scavenging activity, and (d) FRAP assays of non-industrial FWJ under different storage conditions.

Figure 3a reflects the variation in ascorbic acid contents of FWJ under different storage conditions. The initial ascorbic acid content of FWJ was 39.88 µg/mL, which was close to the measured data reported by Rawson et al. (26) and Oliu et al. (27). Overall, the increase in storage time and storage temperature led to a significant reduction in ascorbic acid content in FWJ, especially for juices stored at 37°C which showed a more pronounced downward trend in comparison with 4 and 25°C (*P*<0.05), which was consistent with previous research on changes in ascorbic acid during storage of other fruit juices (23, 25, 28). Ascorbic acid is sensitive to a variety of factors, such as oxygen, pH, heat, water activity, and metal ions (23). The degradation of ascorbic acid during juice storage was mainly due to oxidative degradation; ascorbic acid can be easily oxidized in the presence of oxygen by both enzymatic and non-enzymatic catalysts (24, 29). Oxidative degradation is positively correlated with storage temperature. In addition, the ascorbic acid content of juices at 4, 25, and 37°C decreased by 16.82, 21.14, and 30.50%, respectively, after storage for 4 h, while it decreased by 66.51, 76.67, and 83.90%, respectively, after storage for 24 h.

#### Total polyphenol content

It could be seen from Fig. 3b that the TPC of FWJ stored at 4°C remains basically unchanged (*P*>0.05). Vallverdú-Queralt et al. (30) also found that phenolic compounds were stable in tomato juice during storage for 4°C for 3 months. In addition, the TPC in the juice stored at 25 and 37°C increased significantly within 0–2 h (*P*<0.05), increased gradually from 2 to 12 h, and then dropped sharply during 12–24 h (*P*<0.05). This trend was probably because the dissolution and extractability of polyphenol compounds were relatively lower at 4°C, while it was significantly enhanced when stored at a higher temperature, especially at 37°C. Furthermore, the oxidative degradation of polyphenols was susceptible to occur at 25 and 37°C, while relatively insusceptible to occur at 4°C (30, 31), which well explained the variation trend of TPC in FWJ under different storage conditions. In addition, the decline in TPC in the later stage of storage may be caused by the aggregation of polyphenols with proteins in the juice (14).

#### Antioxidant activity

The positive effects of juices on human health are mainly attributed to the antioxidant substances that they contain and their associated antioxidant activities (32). Fig. 3c, 3d represents the variation in DPPH scavenging activity and FRAP assays of FWJ under different storage conditions. Apparently, a downward trend in DPPH scavenging activity and FRAP assays could be observed during the whole storage period in juices stored at 4°C. However, the two indexes of juices stored at 25 and 37°C showed an upward trend first and then a downward trend. It is not difficult to perceive that the decrease in TPC and ascorbic acid content during storage is reflected by the decrease in DPPH and FRAP antioxidant capacities of FWJ (Fig. 3c, d). On the whole, the variation trend of DPPH scavenging activity and FRAP assays was highly in accordance with the change in TPC. Klimczak et al. (33) have also demonstrated a strong correlation between antioxidant capacity and TPC values during the storage of orange juice.

Considering the above nutritional and functional indicators comprehensively, it can be concluded that fresh juice stored at 4 and 25°C can still retain good nutritional and functional characteristics within 4 h, while juice stored at 37°C can still retain good nutritional and functional characteristics within 2 h.

### Dynamic variation rules of the sensory quality of non-industrial FWJ under different storage conditions

#### Organoleptic evaluation

**Fig. 4:**
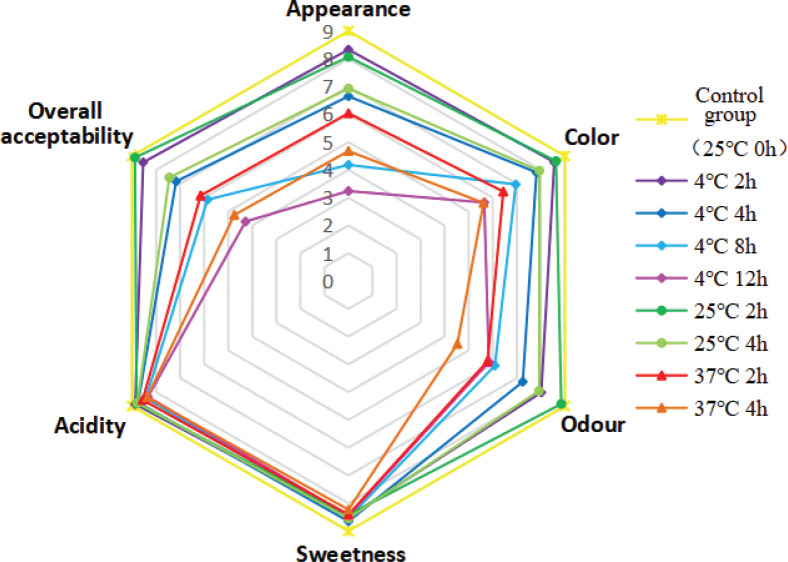
Dynamic change of oranoleptic indicators of FWJ under different storage conditions: (a) spider plot of sensory analysis; (b) color (∆*E*) and lightness (L*); and (c) suspension stability.

The sensory characteristics of any food have an important impact on acceptance or rejection by its consumers. An artificial sensory evaluation test on juice samples that was safe for microbial indicators was performed. The sensory evaluation with respect to appearance, color, odor, sweetness, acidity, and overall acceptability of FWJ under different storage conditions is shown in Fig. 4. It is clear that acidity and sweetness had less effect on the sensory score of watermelon juice. Conversely, the appearance, color, and odor had a greater influence. In addition, it was found that the odor of the juice stored at 25°C was closest to the control group, while the sensory indexes of the juice at 37°C deteriorated with the increase in storage time. In short, the FWJ still had acceptable sensory properties (sensory scores ranging from 7.17 to 8.88) when stored at 4 and 25°C for no more than 4 h.

#### Color analysis

**Fig. 5:**
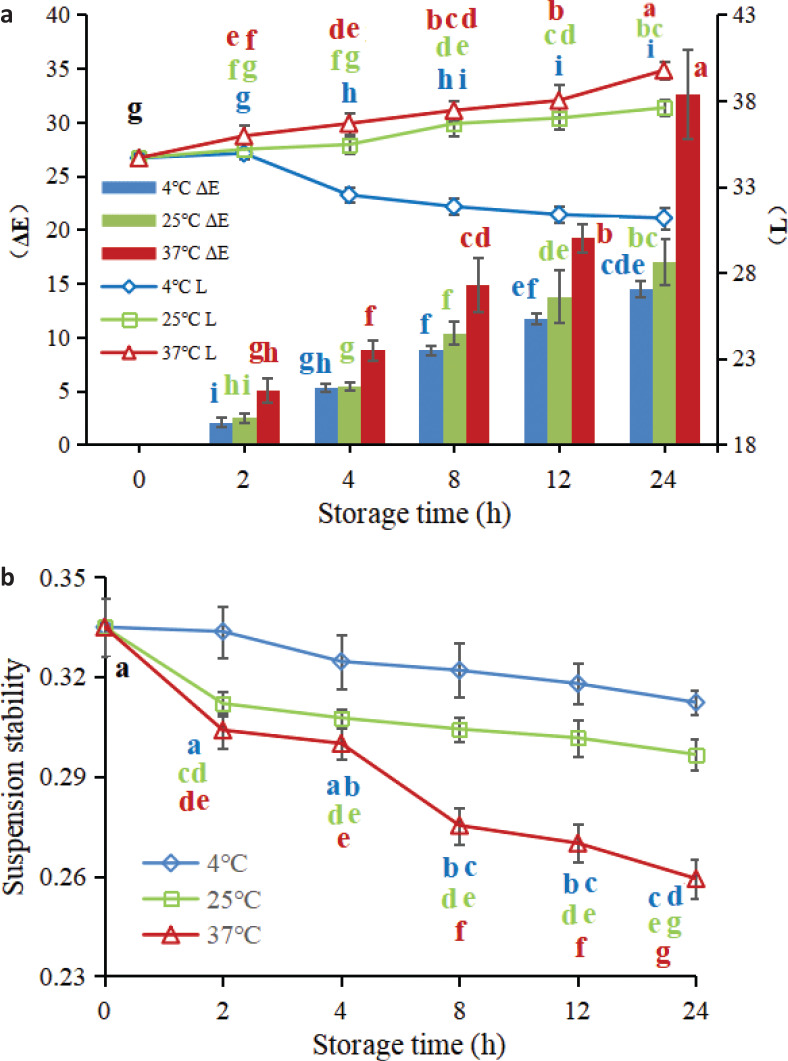
Dynamic changes in the sensory characteristics of non-industrial FWJ under different storage conditions: (a) Color (∆*E*) and lightness (L*); and (b) suspension stability.

Color is also a crucial parameter for the evaluation of juice quality. The ∆*E* value represents the total color difference, while the L* value represents the lightness index. According to Fig. 5a, it is obvious that both storage time and storage temperature had a significant impact on L* and ∆*E*. The ∆*E* value of FWJ under different storage conditions increased with the increase in storage time. Zou et al. (34) and Guan et al. (35) also found similar changes in mulberry juice and mango juice. A noticeable difference can be observed between two colors when the ∆*E* value is greater than 2 (36). Therefore, the visible color change in FWJ could be observed after 2 h of storage. In addition, the ∆*E* value of juice stored at 37°C was significantly higher than that stored at 4 and 25°C (*P*<0.05). During the experiment, it was also noticed that the higher the storage temperature, the more serious the fading phenomenon of FWJ, possibly due to more severe browning reactions occurring at 37°C (35). In addition, when the storage temperature rises, the pigment in the juice is more easily destroyed and degraded, and the polymerization reaction between the pigment and the polyphenol is also intensified (37). This study concluded that higher storage temperature accelerated the color deterioration of the FWJ and further affected the sensory quality of the juice.

In addition, during the whole storage period, the L* value of the juice stored at 25 and 37°C increased gradually. In contrast, the L* value decreased significantly when juice was stored at 4°C (Fig. 5a), which indicated that FWJ stored at 25 and 37°C became brighter gradually, whereas that stored at 4°C was significantly darker. Currently, there is still controversy about the change in the L* value during fruit juice storage. Zou et al. (34) and Gironés-Vilaplana et al. (38) reported that the L* value in mulberry juice and maqui berry and lemon juice tended to increase during the storage period at 4 and 25°C. Conversely, Guan et al. (35) and Calligaris et al. (39) found the L* value of mango juice and banana juice decreased over the entire storage period. The downward trend in the L* value during fruit juice storage has also been reported in other studies (24, 25, 37). Therefore, further research is still needed.

#### Suspension stability

Suspension stability reflects turbidity stability under certain centrifugal forces. It is an important factor affecting the consumer’s acceptance of juice products. Consumers generally do not want the juice to be stratified or emulsified, that is, they want the juice to have stable turbidity. Obviously, with the increase in storage time and temperature, the suspension stability of FWJ showed a significant downward trend (Fig. 5b), and the degree of decrease for 25 and 37°C was more pronounced in comparison with 4°C (*P*<0.05). Hence, it can be concluded that storage at 4°C was beneficial to maintain the suspension stability of FWJ.

Overall, 4°C of storage was beneficial to maintain the suspension stability and original color of FWJ. However, storage at 4°C significantly darkened the FWJ, which also negatively affects the color of the juice. In short, it can be concluded that FWJ still had acceptable sensory properties when stored at 4 and 25°C for no more than 4 h.

### Using an electronic nose to evaluate and discriminate non-industrial FWJ under different storage conditions

**Fig. 6:**
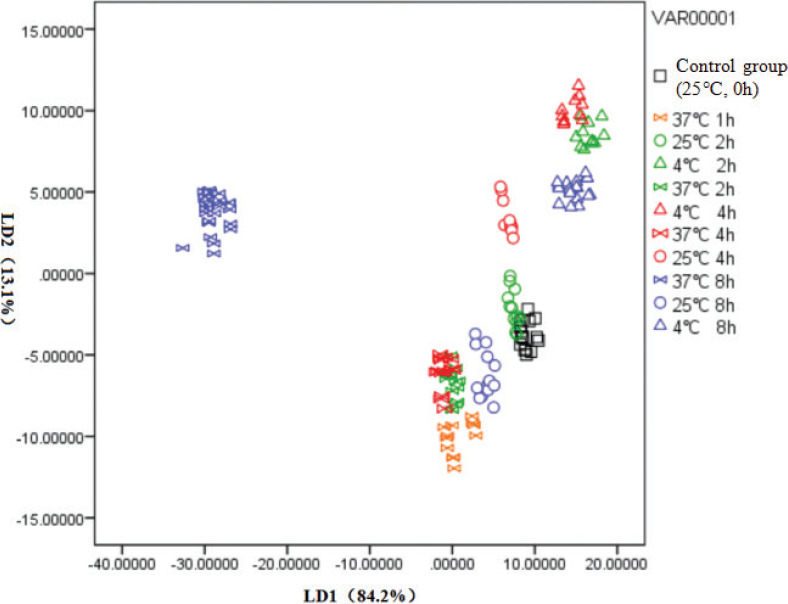
LDA results of non-industrial FWJ under different storage conditions.

To discriminate and classify different juice samples, the average stable signal of each sensor at 56–60 s of an electronic nose was used in this study; all sample data were subjected to linear discriminant analyses (LDAs) (40). Figure 6 depicts the LDA results of different FWJs based on electronic nose response data. The two discriminant functions explained 97.3% of the total variance, including 84.2% by LD1 and 13.1% by LD2. In general, the electronic nose with LDA models can distinguish 11 groups of FWJs under different storage conditions. Although there is some overlap or some sample points are very close, they are not mixed together. Juice samples with different storage conditions showed a regular distribution. It is clear that the FWJ stored at 25°C, especially the juice stored at 25°C for 2 h, was closer to the control group, and its aroma characteristics were closest to the control group. This finding indicated that the aroma characteristics of FWJ stored at 25°C for 2 h were basically the same as those of the control group. This result is consistent with the previous results of artificial sensory evaluation (Fig. 4). In addition, the distribution of juice samples stored at 4 and 37°C was far from the control samples, indicating the odor characteristics of juices stored at 4 or 37°C changed significantly. Consequently, the electronic nose could be used as a powerful alternative approach to evaluate and discriminate FWJs under different storage conditions due to its high sensitivity and objectivity.

#### Using GC–MS to investigate the change in volatile components of non-industrial FWJ under different storage conditions

Identification of volatile components and the relationship between their relative contents may be a useful means for identifying varieties (41), adulterations (40), and freshness of fruit juices. Based on the results of artificial sensory evaluation (Fig. 4) and an electronic nose (Fig. 6), the odor characteristics of FWJ changed significantly under different storage conditions. Thus, an electronic nose can effectively distinguish different fruit juice samples (Fig. 5). To further explore the impacts of storage time and temperature on volatile components in FWJ, GC–MS was used to qualitatively and quantitatively analyze and compare the changes in volatile components in FWJ under different storage conditions. The results are presented in Table 1.

**Table 1:** The variety and concentration of volatile compounds in nonindustrial FWJ under different storage conditions

No.	Compounds	Concentration(µg/L)							CAS
		Control group	4°C (2 h)	4°C (8 h)	25°C (2 h)	25°C (8 h)	37°C (2 h)	37°C (8 h)	
1	Carbamic acid, monoammonium salt	22.21±1.06a	10.34±0.45b	—	9.86±0.32b	—	—	—	1111-78-0
2	Carbon dioxide	**—**	—	16.55±1.05a	—	8.42±0.53c	10.40±0.45b	9.49±0.35b	124-38-9
3	Acetaldehyde	18.95±0.91e	28.40±1.43c	69.40±3.13a	28.60±1.39c	31.17±1.09c	25.21±1.18d	14.99±0.68f	75-07-0
4	Ethanol	153.17±3.18d	169.99±2.05c	373.45±3.35a	152.72±2.86d	173.37±1.22c	170.54±2.34c	195.09±2.47b	64-17-5
5	Acetone	—	—	—	—	—	—	3.96±0.024a	67-64-1
6	Dimethyl sulfide	—	—	—	—	—	—	25.18±0.076a	75-18-3
7	Dimethyl-diazene	8.20±0.21b	4.88±0.043c	14.44±0.96a	7.03±0.25b	4.80±0.18c	4.85±0.11c	—	503-28-6
8	Pentane	1.84±0.016d	—	25.89±0.43a	10.59±0.53b	—	9.12±0.18c	—	109-66-0
9	2-Methylpentane	2.26±0.012e	7.23±0.26b	13.01±0.38a	3.09±0.046d	4.94±0.24c	6.31±0.30b		107-83-5
10	3-Methylpentane	1.62±0.018d	4.08±0.13b	8.84±0.26a	1.99±0.022d	3.26±0.12c	3.44±0.086c	4.26±0.095b	96-14-0
11	Sec-Butyl nitrite	—	52.67±1.69b	105.68±3.23a	—	—	—	37.24±1.34c	924-43-6
12	Ethyl acetate	38.96±1.53a	—	—	37.23±1.26a	—	—	—	141-78-6
13	2-Methylfuran	13.88±0.48d	—	28.28±1.16a	14.14±0.35d	—	19.26±0.52c	20.27±0.93b	534-22-5
14	3-Methylfuran	**—**	17.01±0.88a	—	—	14.37±0.43b	—	—	930-27-8
15	Tetrahydrofuran	0.81±0.014e	2.41±0.022c	5.97±0.13a	2.04±0.098d	2.42±0.083c	2.88±0.095b	2.18±0.063d	109-99-9
16	Methylcyclopentane	1.19±0.026e	3.42±0.12b	6.02±0.14a	1.15±0.035e	2.74±0.041c	2.89±0.087c	2.54±0.73d	96-37-7
17	3-Methyl-butanal	1.10±0.030e	4.30±0.12c	16.26±0.43a	1.62±0.089e	6.52±0.33b	4.57±0.29c	2.40±0.085d	590-86-3
18	2-Methyl-butanal	**—**	1.12±0.031d	5.04±0.43a	1.31±0.026d	2.45±0.092b	2.04±0.066c	2.37±0.078b	96-17-3
19	1-Penten-3-ol	2.73±0.042c	3.66±0.22b	7.64±0.50a	3.33±0.043c	3.52±0.092b	3.60±0.16b	3.14±0.088c	616-25-1
20	Acetoin	—	—	—	—	—	—	33.29±1.12a	513-86-0
21	Pentanal	2.84±0.075b	—	5.82±0.19a	—	—	—	—	110-62-3
22	Dimethyl silanediol	36.84±1.42b	20.74±0.96c	52.00±1.94a	21.87±0.92c	18.49±0.42d	20.79±0.87d	—	1066-42-8
23	3-Methyl-1-Butanol	—	—	5.11±0.19b	3.46±0.072c	3.72±0.044c	—	8.47±0.32a	123-51-3
24	(E)-2-Pentenal	8.47±0.28b	7.44±0.24c	10.45±0.42a	6.34±0.20d	4.62±0.14e	6.21±0.19d		1576-87-0
25	1-Pentanol	2.20±0.089e	2.64±0.076d	6.60±0.52a	2.50±0.11d	3.45±0.095b	3.04±0.082c	3.42±0.094b	71-41-0
26	3-Hexenal	9.54±0.42b	5.83±0.25c	11.45±0.39a	8.39±0.092b	2.63±0.046e	—	—	4440-65-7
27	Hexanal	229.88±3.92a	134.83±2.86c	124.29±4.24c	242.65±1.75a	126.15±2.34c	117.72±2.27d	6.14±0.19g	66-25-1
28	Nitrocyclopentane	3.33±0.074a	1.36±0.018c	3.01±0.024a	2.43±0.014b	0.88±0.012d	—	—	2562-38-1
29	1-Octene	9.37±0.096a	4.11±0.17d	7.43± 0.28b	6.37±0.087c	2.45±0.072e	2.51±0.043e	—	111-66-0
30	Octane	—	—	4.72±0.066a	—	2.04±0.028b	1.30±0.019d	1.69±0.035c	111-65-9
31	Hexamethyl-cyclotrisiloxane	7.05±0.12b	6.79±0.15b	15.72±0.24a	6.01±0.097c	6.33±0.11bc	6.10±0.099c	5.19±0.074d	541-05-9
32	(E)-2-Hexenal	6.53±0.11a	4.00±0.072c	—	7.34±0.045a	4.68±0.063b	3.67±0.036c	—	85761-70-2
33	(Z)-3-Hexen-1-ol	2.63±0.092d	4.27±0.13c	8.76± 0.39a	2.94±0.27d	5.43±0.16b	5.35±0.35b	5.70±0.23b	33467-73-1
34	1-Hexanol	12.63±0.42d	26.57±1.04c	57.83±1.96a	13.11±1.15d	38.08±1.22b	32.46±1.34c	51.70±1.78a	111-27-3
35	Heptanal	5.28±0.17b	2.94±0.025d	6.44± 0.32a	5.00±0.092b	3.31± 0.086c	2.85±0.042d	—	111-71-7
36	Methoxy-phenyl-oxime	13.22±0.45a	5.60±0.34c	10.54±0.42b	5.76±0.66c	4.66±0.072d	5.17±0.28c	4.07±0.32d	No data
37	(Z)-2-Heptenal	5.65±0.21c	4.04±0.19d	11.40±0.35a	6.88±0.12c	5.13±0.38c	4.39±0.27d	1.50±0.024f	57266-86-1
38	6-Methyl-5-hepten-2-one	—	—	—	17.42±0.24c	21.49±0.79b	21.13±0.62b	26.84±1.15a	110-93-0
39	1-Octen-3-one	1.07±0.019a	—	—	1.04±0.015a	—	—	—	4312-99-6
40	1-Octen-3-ol	12.15±0.22c	12.78±0.39c	31.11±1.15a	15.91±0.22c	—	—	—	3391-86-4
41	3-Octanone	2.06±0.044d	2.36±0.051d	5.84±0.076a	2.24±0.083d	2.92±0.072bc	2.43±0.063d	3.03±0.032b	106-68-3
42	2-Octanone	6.61±0.26c	6.18±0.13c	13.10±0.38a	6.53±0.22c	7.31±0.34b	5.66±0.27d	7.17±0.46b	111-13-7
43	2-Pentylfuran	26.15±0.49e	38.35±0.92d	83.60±1.55c	41.11±1.87d	76.44±1.76c	83.56±1.85c	169.70±2.92a	3777-69-3
44	2,2-dimethyl-decane	1.88±0.022a	—	1.58±0.008b	—	0.88±0.012c	1.95±0.015a	—	17302-37-3
45	Octamethyl-cyclotetrasiloxane	75.19±0.95e	111.71±1.86c	207.68±3.04a	67.07±1.74e	102.18±2.13cd	106.34±1.69c	98.71±2.42d	556-67-2
46	3-Ethyl-2-methyl-1,3-Hexadiene	5.85±0.067b	3.91±0.045d	8.62±0.072a	5.70±0.088b	5.08±0.017c	4.94±0.025c	—	61142-36-7
47	(Z)-2-Decene	—	—	—	—	—	25.35a	—	20348-51-0
48	Cis-3-Decene	13.63±0.32d	19.4±0.58c	45.63±0.92a	23.78±0.75b	21.39±0.66bc	—	16.90±0.52d	19398-86-8
49	1-Ethyl-1-methyl-cyclopentane	1.85±0.012a	1.68±0.015b	—	1.53±0.006c	—	—	—	16747-50-5
50	D-Limonene	15.47±0.29c	13.24±0.28d	39.85±0.72a	18.66±0.64b	17.80±0.81b	13.24±0.29d	13.68±0.45d	5989-27-5
51	(E)-2-Octenal	11.73±0.26b	8.54±0.14d	21.57±0.62a	13.22±0.18b	9.26±0.28c	7.38±0.32e	3.45±0.073f	2548-87-0
52	(E)-2-Octen-1-ol	2.13±0.016d	2.36±0.021c	4.05±0.034a	2.40±0.032c	2.70±0.025c	2.49±0.036c	2.49±0.062c	18409-17-1
53	1-Octanol	2.37±0.092e	5.42±0.041d	9.14±0.067a	2.83±0.053e	6.36±0.032c	5.67±0.044d	6.87±0.055b	111-87-5
54	(E)-4-Nonenal	127.65±3.95a	112.02±2.75b	97.07±1.64c	86.95±2.51d	29.29±1.02f	45.75±1.77e	15.24±0.39g	2277-16-9
55	3,3-dimethyl-1-Octene	—	—	—	—	—	12.66±0.085a	—	74511-51-6
56	Cyclooctanecarboxaldehyde	34.28±0.97a	25.83±0.82b	35.44±1.04a	16.58±0.058c	10.93±0.074d	—	5.32±0.069e	6688-11-5
57	(Z)-6-Nonenal	223.04±2.88a	36.64±1.04f	54.91±1.36e	198.35±1.25ab	18.02±0.43g	19.17±0.52g	6.31±0.081h	2277-19-2
58	Nonanal	429.27±4.96a	83.43±2.79e	167.42±3.55d	374.98± 2.45b	40.43±0.63f	43.34±0.74f	22.37±0.85g	124-19-6
59	Undecane	1.75±0.012b	1.37±0.023c	1.95±0.032a	0.59±0.006e	0.68±0.005de	1.31±0.024c	0.79±0.027d	1120-21-4
60	2,6-Nonadien-1-ol	12.93±0.33a	9.10±0.28b	12.39±0.39a	6.12±0.26c	4.14±0.17d	4.82±0.099d	—	7786-44-9
61	(E)-2-Nonenal	27.97±0.92a	20.13±0.75b	28.98±1.08a	21.86±0.54b	9.53±0.043d	9.28±0.26d	2.83±0.088e	18829-56-6
62	(E,Z)-2,6-Nonadienal	203.74±3.55a	127.83±1.26c	119.51±2.09c	195.85±2.33a	101.28±0.92d	87.64±2.27e	24.51±0.65f	557-48-2
63	(Z)-3-Nonen-1-ol	7.55±0.096d	1006.28±15.07a	992.26±6.09a	989.86±12.25a	1039.99±13.28a	858.07±8.44b	792.18±9.78c	10340-23-5
64	(E)- 3-Nonen-ol	—	14.20±0.12c	—	—	17.76±0.32ab	—	18.18±0.46a	10339-61-4
65	(6Z)-Nonen-1-ol	—	74.40±1.04c	126.89±1.87a	87.26±0.95b	90.79±1.36b	—	—	35854-86-5
66	(6E)-Nonen-1-ol	—	—	—	—	—	86.17±1.82b	97.68±2.05a	40709-05-5
67	(E)-3-Decen-1-ol	1015.75±9.82a	—	—	—	—	—	—	69093-74-9
68	(E)-2-Nonen-1-ol	7.54±0.093b	—	—	9.77±0.12a	—	2.49 ± 0.012d	—	31502-14-4
69	1-Nonanol	19.82 ± 0.088f	183.10±2.56c	334.56±2.92a	22.30±0.42f	202.27±1.69b	179.45±3.74c	185.35±2.51c	143-08-8
70	Decamethyl-cyclopentasiloxane	181.32±2.72d	192.88±2.44c	426.27±4.86a	114.47±2.37e	201.32±3.58c	207.57±3.05c	169.18±1.93d	541-02-6
71	(E,E)-2,4-Nonadienal	5.70±0.070a	5.22±0.052b	5.25±0.036b	4.85±0.018c	2.20±0.013f	4.32±0.025d	—	5910-87-2
72	(E)-4-Decenal	1.25±0.024a	—	—	1.00±0.015b	—	—	—	21662-09-9
73	(E,Z)-3,6-Nonadien-1-ol	1.69±0.017d	1.94±0.025bc	2.72±0.022a	1.58±0.037d	1.81±0.032c	1.88±0.028c	2.03±0.019b	56805-23-3
74	Decanal	1.45±0.014a	0.56±0.005c	—	1.09±0.009b	0.44±0.006c	—	—	112-31-2
75	Dodecamethyl-cyclohexasiloxane	0.57±0.015f	53.36±0.95c	131.62±1.34a	31.92 ± 0.84e	63.35±1.86b	49.42±1.07d	—	540-97-6
76	2-Methyl-propanoicacid, 3-hydroxy-2,2,4-trimethylpentyl ester	1.29±0.027e	2.17±0.038c	5.71±0.083a	1.80±0.055d	2.29±0.033c	2.51±0.025b	—	74367-34-3
77	6,10-dimethyl-5,9-Undecadien-2-one	1.56±0.042e	1.75±0.056d	5.42±0.042a	2.70±0.036c	3.34±0.064b	2.64±0.031c	—	689-67-8
78	2,4-Di-tert-butylphenol		4.36±0.11d	9.75±0.23a	4.80±0.094c	5.11±0.045b	3.94±0.037e	—	96-76-4
79	3,5-bis(1,1-dimethylethyl)- Phenol	1.16±0.022b	—	—	3.73±0.042a	—	—	—	1138-52-9
80	Hexanoic acid, 3,5,5-trimethyl-, 2-ethylhexyl ester	1.97±0.035a	—	—	0.54±0.012c	—	—	—	70969-70-9

Note: Volatiles were shown according to their order of appearance in the chromatogram on DB-Wax column.

— represents not detected. Different letters in the same line indicate significant differences (Duncan’s test: *P* < 0.05, performed by DPS software (version 7.55, China).

In total, 80 kinds of volatile components were identified from seven groups of FWJs under different storage conditions: 18 species of alcohols, 16 species of aldehydes, 14 species of alkanes, 7 species of ketones and alkenes, 5 species of esters, 4 species of furans, 2 species of sulfide and phenols, 1 carboxylic acid, 1 nitride, and 3 unknown compounds. The volatile compounds and their concentrations were significantly different in different sample groups. A total of 65 volatile compounds were observed in the control group (25°C, 0 h). Among them, ethanol (4.93%), hexanal (7.40%), (E)-4-nonenal (4.11%), (Z)-6-nonenal (7.18%), nonanal (13.82%), (E,Z)-2,6-nonadienal (6.56%), (E)-3-decen-1-ol (32.69%), and decamethylcyclopentasiloxane (5.84%) were found to be the eight major compounds, accounting for 82.53% of the total content of volatile compounds. Similarly, Liu et al. (4) identified 55 compounds from watermelon juice using SPME GC–MS/O. Hexanal, nonanal, (E)-2-nonenal, (E,Z)-2,6-nonadienal, (Z)-3-nonen-1-ol, and (E,Z)-3,6-nonadien-1-ol showed to be the main aroma components of watermelon juice. However, in the investigation by Lea et al. (42), in which 59 compounds were identified from watermelon, hexanal, Z-6-nonenal, nonanal, (E,Z)-2,6-nonadienal, (E,Z)-3,6-nonadien-1-ol, and E-2-nonenal accounted for 77.3–81.6% of the watermelon volatiles. Although some volatile compounds are not exactly the same as previously reported, C_9_ alcohols and aldehydes, such as hexanal, nonanal, (Z)-6-nonenal, (E,Z)-2,6-nonadienal, (E,Z)-3,6-nonadien-1-ol, and (Z)-3-Nonen-1-ol, have been confirmed to be the typical volatile compounds in watermelon by much research (4, 42, 43). The differences in the volatile substances among these studies were probably attributed to watermelon variety and origin, ripeness, extraction method, absorbing material, chromatographic column polarity used, and so on. C_9_ alcohol and aldehyde, which are generally considered as the products of oxidation and pyrolysis of polyunsaturated fatty acids (PUFA), such as linoleic acid and linolenic acid, under the action of lipid oxidase, hydrogen peroxide lyase, and alcohol dehydrogenase, were the main aroma substances in watermelon juice (4). It is remarkable that (E)-3-decen-1-ol (32.69%) was the most abundant flavor substance in the control group. However, at any storage temperature, for a storage period of less than 2 h, a large amount of (E)-3-decen-1-ol was converted to (Z)-3-nonen-1-ol, probably due to the action of isomerase and oxidase in the oxidative decomposition pathway of linoleic acid or linolenic acid (4). In addition, other aldehydes in watermelon juice, such as hexanal, (E)-4-nonenal, (Z)-6-nonenal, nonanal, and (E,Z)-2,6-nonadienal, decreased significantly throughout storage. In particular, (E)-4-nonenal showed a significant negative correlation with storage time and temperature.

From Table 1, it is clear that 65 and 66 types of volatile substances were identified in the control group and the 25°C, 2 h group, respectively. In comparison with the control group, both the composition and concentration of volatile substances in the 25°C, 2 h group were the closest to the control group. Specifically, the 25°C, 2 h group and control group contained 61 identical volatile substances, 27 kinds of which had no significant difference in their concentrations (*P*<0.05). More importantly, among the 8 kinds of major flavor substances in FWJ, the concentration of ethanol, hexanal, Z-6-nonenal, and (E,Z)-2,6-nonadienal showed no significant difference between the two groups (*P*<0.05). Thus, the aroma characteristics of the 25°C, 2 h group were the closest to the control group. This finding also verifies the results of the electronic nose (Fig. 6). In contrast, compared with control group, only 57 and 48 types of volatile substances were identified in the 37°C, 2 h group and 37°C, 8 h group, respectively, and the content of typical volatile compounds, such as hexanal, (E)-4-nonenal, (Z)-6-nonenal, nonanal, and (E,Z)-2,6-nonadienal, declined rapidly with the storage duration. In addition, juices stored at 37°C produced six new volatile substances (content ≥20 µg/L): dimethyl sulfide, acetoin, 6-methyl-5-hepten-2-one, (Z)-2-decene, 3,3-dimethyl-1-octene, and (6E)-nonen-1-ol. The content of 2-pentylfuran increased sharply. In addition, juices stored at 4°C mainly produced two new volatile substances (content ≥20 µg/L): sec-butyl nitrite and (6Z)-nonen-1-ol. The content of ethanol, 1-hexanol, 2-pentylfuran, octamethylcyclotetrasiloxane, 1-nonanol, and dodecamethylcyclohexasiloxane increased significantly.

Overall, the volatile components of FWJ changed significantly under different storage conditions. Compared with juices stored at 4 and 37°C, the odor characteristics of FWJ stored at 25°C for less than 2 h, which were closest to the control group, were the best. However, when the storage time exceeded 2 h, the volatile substances were well preserved at 4°C on the whole. However, storage of juices at 37°C not only reduced the types of volatile components but also caused the escape of typical aroma components and produced some unpleasant odors, such as that from dimethyl sulfide.

## Conclusion

People often ask if overnight non-industrial juice is still drinkable. In the present study, the quality change rules, including microbial safety quality, nutritional quality, and sensory quality, of non-industrial FWJ under different storage conditions were systematically investigated. In general, 4°C storage was most beneficial to ensure the microbiological safety and prolong the time range for safe drinking. The time range of microbial-safe drinking of the FWJ stored at 4, 25, and 37°C was within 12, 4, and 4 h, respectively. Moreover, the increase in storage time and storage temperature led to a significant reduction in ascorbic acid content in FWJ. The TPC of juices stored at 4°C remained basically unchanged, while that of juices stored at 25 and 37°C first increased and then decreased, and the antioxidant activity was highly in accordance with the change in TPC. Overall, with regard to the nutritional and functional indicators, fresh juice stored at 4 and 25°C can still retain good nutritional and functional characteristics within 4 h, while juice stored at 37°C can do so within 2 h. For the sensory quality, 4°C storage was beneficial to maintain the suspension stability and original color of non-industrial FWJ. Non-industrial FWJ stored at 25°C for less than 2 h demonstrated the best odor characteristics, which were closest to the control group. In short, it can be concluded that non-industrial FWJ still had acceptable sensory properties when stored at 4 and 25°C for no more than 4 h. Based on the results of nutritional and sensory quality, a conclusion could be drawn that the drinking time range of nonindustrial FWJ with good quality was within 2 h when stored at 4 or 25°C, and the nonindustrial FWJ still had acceptable nutritional and sensory quality when stored at 4 or 25°C for no more than 4 h. Furthermore, an electronic nose and GC–MS can effectively distinguish and identify the changes in volatile components in FWJ under different storage conditions. Thus, the combination of an electronic nose and GC–MS can be used as a new method to evaluate the shelf life and the quality of nonindustrial FWJ. Therefore, in terms of the juice microbial safety quality, nonindustrial FWJ stored at 4°C for 12 h is still drinkable, which means that overnight juice is still drinkable. However, with regard to the nutritional quality, after storage at 4°C for 12 h, the ascorbic acid content of juice decreased by approximately 36%. The sensory quality also decreased significantly. Obviously, the juice was no longer of good quality at this time.
